# A laboratory-focussed desk review of health systems in Uganda, Kenya, and the UK to respond to current and future pandemics

**DOI:** 10.7189/jogh.15.04194

**Published:** 2025-08-29

**Authors:** Winters Muttamba, Bernadette O’Hare, Andrew Ramsay, Vibhor Saxena, Bruce Kirenga, Wilber Sabiiti

**Affiliations:** 1 University of St Andrews, St Andrews, UK; 2 Makerere University Lung Institute, Kampala, Uganda; 3 Interdisciplinary Consortium for Epidemics Research, Kampala, Uganda

## Abstract

**Background:**

Laboratory systems play a crucial role in managing diseases effectively, and the COVID-19 pandemic serves as a prime example. The pandemic underscored the need to make laboratory health systems more resilient and robust to respond to future pandemics.

**Methods:**

We conducted a desk review guided by the six World Health Organization health system building blocks (health service delivery, health financing, medical products, vaccines, and technologies, human resources for health, governance, and health information systems).

**Results:**

The three countries' strengths include health information systems, well-established reference laboratories, mobile and community-level testing, a vibrant private laboratory sector in Uganda and Kenya, and a growing private sector in the UK. In Uganda and Kenya, there are laboratory connectivity solutions for molecular diagnostics, multi-disease testing platforms and specimen referral systems, while in the UK, there are hub-and-spoke networks. Weaknesses in Uganda and Kenya include vertical laboratory systems strengthening, ill-equipped laboratories, constrained and inequitable distribution of laboratory human resources, and limited data use. In the UK, there is chronic underfunding and undervaluing of disciplines supporting infection testing, microbiology and virology.

**Conclusions:**

The growing contribution of the private sector in the three countries and the deployment of multi-disease testing platforms should be supported, given the advantage of shared financial costs in the face of chronic underfunding for laboratory systems.

COVID-19 is now an established and ongoing disease and no longer considered a public health emergency of international concern by the World Health Organization (WHO) [1]. Despite this announcement from the WHO, up to 34 812 cases are reported globally each week. [2].

The global community was ill-prepared to respond to the pandemic when it began. In the early days of the pandemic, and in the absence of proven pharmaceutical interventions, management was largely non pharmacological and consisted of isolation of cases, quarantine, physical distancing, school and workplace closures and masking [3,4] Later, drug repurposing – where existing drugs are used for a new treatment that was not indicated before, was deployed. Several drugs, such as hydroxychloroquine, ritonavir/lopinavir, favipiravir, remdesivir, ivermectin, dexamethasone, immunotherapeutic molecules (*e.g.* tocilizumab, mavrilimumab, baricitinib, and interferons) were repurposed [5]. Later, antivirals (*e.g.* Paxlovid, molnupiravir) were deployed, which demonstrated efficacy and safety [6]. Vaccination later emerged as a powerful medical countermeasure against COVID-19 and is believed to have averted 2.5 million deaths [7].

Despite the availability of pharmacological and non-pharmacological measures to control the COVID-19 pandemic, the pivotal role of laboratory systems in making the etiological diagnosis, patient monitoring, and surveillance [8-10] cannot be overemphasised. Further, the International Health Regulations (IHR) 2005 require countries to develop robust laboratory capacities.

It has been reported that lack of laboratory reagents and supplies and limited laboratory capacity increased the COVID-19 testing turn-around time [11]. Studies have documented deficiencies in COVID-19 testing, with many countries lacking testing resources such as testing kits and personnel [12]. Other challenges reported included inability to sustain a skilled laboratory workforce and lack of dedicated supply chain for laboratories [13]. It is believed the testing challenges affected the response effectiveness in several countries, as limited testing affects the other activities in the response cascade such as contact tracing, isolation and reporting [14]. In Africa, limited testing could have led to under estimation of the burden of the pandemic [15]. In a bid to meet the shortfalls in testing, the laboratories in the private sector were brought on board to supplement government efforts in both development of test kits and testing [16-18].

The COVID-19 pandemic underscored the need to make healthcare systems more resilient and robust for future pandemics. The laboratory plays an integral part in building this resilience; however, we postulate there might be weaknesses that need to be addressed and strengths that need to be consolidated as part of building this resilience. We thus undertook a laboratory focused desk review of health systems of Uganda (low income), Kenya (middle income) and the UK (high income) to reveal strengths and weaknesses of laboratory systems and inform laboratory systems strengthening efforts to counter future threats.

## METHODS

### Design

We conducted a desk review of the healthcare systems of Uganda, Kenya, and the UK to assess each country's laboratory system. We reviewed the laboratory systems based on the six WHO building blocks of a healthcare system, and were further guided by the USAID module on assessing the health systems and their core functions [19]. The USAID module on evaluation of health systems prioritises key weakness areas and supports identifying potential recommendations for interventions. The WHO building blocks include: health service delivery, health financing, medical products, vaccines, technologies, human resources for health, governance, and health information systems. The available literature enabled the assessment of building blocks for each country.

### Eligibility criteria

We included literature written in English up to 1 August 2023. The documents reviewed included policies, strategic plans, reports, guidelines, and circulars with relevant information on potential strengths and weaknesses of the laboratory systems.

### Information sources

The documents were accessed from multiple sources, including websites of relevant ministries, departments, and agencies in the study countries and regional and global organisations such as the World Bank, WHO, and the Africa Centres for Disease Control and Prevention (Africa CDC). All literature that included opinion pieces and recommendations without evidence of implementation or lacked clarity was excluded.

### Search strategies

A search was undertaken using PubMed, Embase, Medline, Google Scholar, and Google's search engine, with search words covering each of the six building blocks. The search terms under each building block were as follows:

− Health service delivery: health systems; health facilities; healthcare workers; healthcare access; health facility density; laboratory coverage; laboratory services; laboratory health systems; laboratory health workforce.
− Health financing: health budget; health funding; laboratory funding; out of pocket health expenditure; public health expenditure.
− Medical products, vaccines and technologies: essential medicines/essential laboratory commodities- selection, procurement, distribution; diagnostics.
− Human resources for health: health worker training; laboratory health workers; health training institutions.
− Governance: laboratory/health worker policy; laboratory policy; health policies; health sector regulation; legislation; private health sector; community health systems.
− Health information systems: data collection systems; digital health systems; laboratory information systems; health data quality; health data use.

These search terms were linked to each country using the following: ‘Uganda’ OR ‘Kenya’ OR ‘United Kingdom’ OR ‘Scotland’ OR ‘Wales’ OR ‘England’ OR ‘Northern Ireland’.

### Selection process

The general screen included all documents relevant to the laboratory systems in the study countries. Further refinement considered only documents offering information on the potential strengths and weaknesses of the laboratory systems. The selection was carried out by the first author, was then reviewed by all the co-authors, after which agreement was reached on the list of documents to be included.

### Data items

This review was largely qualitative, and qualitative statements and aspects pointing to potential strengths and weaknesses of the laboratory systems in the study countries were sought.

### Risk of bias assessment

As this was mainly a qualitative desk review, assessment of bias focused mainly on the documents' source, content and context. Most of the documents were government-level documents obtained from credible sources, *i.e.* websites of the relevant ministries, departments and agencies, and well-recognised global institutions (World Bank, WHO, and Africa CDC). In terms of context, the documents were considered in response to realising specific outcomes, such as health outcomes and were informed by baseline assessments. Contextually, the documents reviewed were aligned to global aspirations/instruments. The websites included were credible, with some belonging to government institutions, while the articles were all published in peer-reviewed journals.

### Analysis

We employed a narrative analysis, which is a more suitable approach when statistical meta-analysis or other specialised analyses are not feasible [20]. A narrative analysis depends on words and texts to summarise and clarify the findings of the synthesis.

## RESULTS

**Figure 1:**
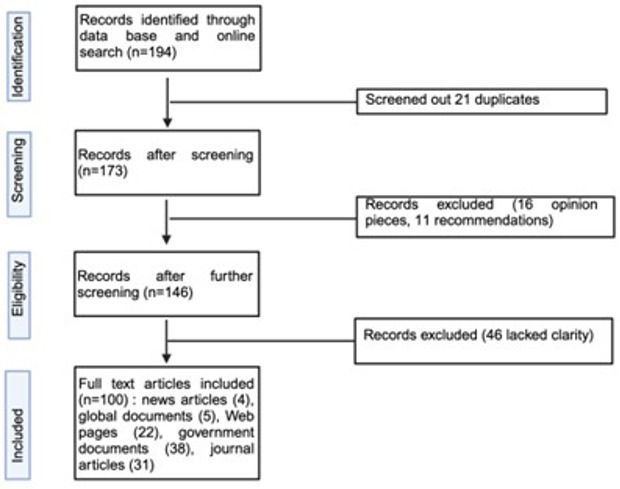
Flow diagram of the literature screening process.

The desk review yielded 194 sources, of which 100 were relevant to this study (highlighting potential strengths and weaknesses of the laboratory systems) and were thus considered. They included 38 documents from government ministries, departments, and agencies, five global reports, 22 webpages, four news articles, and 31 peer-reviewed journal articles (**Figure 1**). The findings are organised around the six WHO health system building blocks (**Table 1**).

**Table 1:** Summary of assessment findings

	Uganda	Kenya	UK
**Service delivery**	There are national reference laboratories with capacity for surveillance, early detection and control of epidemics; however, these are located in the capital [21]. Gradual increase in number of health facilities, 99% of which are able to do basic tests, but few are able to do sophisticated tests [22]. There is a vibrant private health sector that supplements public facilities in provision of laboratory services [23]. There is equipment breakdown, lack of equipment, reagents and supplies which affect laboratory service delivery [24,25]. There are strategies to increase access to testing *e.g.* community level testing, specimen referral systems and mobile laboratory testing [26]. There is an accreditation system to improve the quality of laboratory services [27].	Increased access to health facilities (87.9% of the population live within a 5km distance of a health facility) [28]. There are national reference laboratories with capacity to do surveillance, early detection and control of epidemics [29]. There are two mobile laboratories deployed during disease outbreaks [30]. There is a centre of excellence for laboratory equipment management [31]. There is a specimen referral system to link laboratories that supports diagnosis of 8 zoonotic and 18 dangerous pathogens [32,33]. A DLSP has strengthened the laboratory to undertake disease surveillance and support outbreak investigation [32]. There is an accreditation system to improve the quality and competency of laboratories [34].	Pathology services are involved in 70% of all diagnoses made in the NHS [35]. Quality of laboratories is ensured by UK Accreditation Service which registers and accredits laboratories [36]. There are hub and spoke networks established to increase access to tests [37]. The National Pathology Exchange service facilitates the transfer of test requests and pathology results from any laboratory to any laboratory in the shortest term [35]. There is a national pathology quality assurance dashboard that gives an indication of the performance of pathology services [38].
**Financing**	The budget allocation to health is below 15%, with overreliance on development partners. The country avails supplementary funding during disease outbreaks [39]. Out-of-pocket expenditures are very high (18%). There is vertical programming for laboratory strengthening by development partners [40]. Fewer projects are available for laboratory strengthening [40]. The performance-based financing has been piloted in some projects [41-43].	The budget allocation to health is below 15%. There is less reliance on donor support, with the health budget heavily funded by the government [44]. Out-of-pocket expenditure is higher than the global average but lower than the average for lower middle-income countries. There is a national health insurance scheme to protect citizens against out-of-pocket expenditures (1 in 4 persons covered by insurance) [45]. The absorption rates of funds are still low [46]. There is donor funding for laboratory strengthening projects (*e.g.* the DLSP is heavily CDC funded) [32].	Health expenditure has gradually increased [47,48]. Out-of-pocket expenditure has gradually increased. The government’s commitment to increase funding has been reactionary including borrowing when there is increased demand during health outbreaks [49]. The taxation systems allow for redistribution of health care costs from the rich to the poor, but not sustainable in health emergencies [50]. There is chronic underfunding and undervaluing of disciplines that support testing of infections has been noted [51].
**Medical, devices, drugs, vaccines, and technologies**	Funding for EMHS is heavily donor reliant [52]. Funding for laboratory commodities has not increased to match demand [53]. There are strategies to support local production of EMHS (*e.g.* tax holidays, promoted by the Buy Uganda Build Uganda initiative) [52]. There is a dual push-pull delivery system though this has been blamed for stock outs and expiries [54]. Supply chain systems are largely manual, leading to errors [55]. There is shortage of trained staff to support chain supply systems [55]. There is sufficient inclusion of laboratory items on EMHS list [56]. There is a standard test menu, techniques and supplies list [57]. Laboratory performance is affected by stockouts and equipment breakdown [24].	The pull delivery system is used with counties making requests to KEMSA [58,59]. Distribution of supplies is largely automated, which improves efficiency [58]. Guidelines for emergency procurements are lacking [60]. There is an essential medical laboratory commodity list developed in 2014 [61]. There is a funding gap for laboratory supplies (only 6% of the required 24% of the budget is allocated) [28]. There are shortages of laboratory supplies in health facilities [28].	There are frameworks for assessment of new pharmaceuticals and technologies [50]. Spending on R&D has increased gradually [62]. There is lack of transparency in the way procurements were done during emergencies [63]. There is low stockpile for laboratory supplies [51].
**Human resources**	There is a department of health education and training within the Ministry of Education [64]. There are quality gaps in health training institutions [65]. There is minimal increase in number of human resources available for health (staffing level is 78%) [66]. The workforce coverage is inequitable, with regional differences in staffing [66]. There is reactionary hiring of personnel during disease outbreaks [66]. There is understaffing for laboratory workers in public health facilities [67]. The laboratory staff positions are 97% filled in the private not for profit health facilities [67]. Structures to absorb some laboratory cadres from training institutions are missing [68].	There are supportive frameworks/infrastructure for human resources for health such as iHRIS and advisory boards [69]. Health worker density is low with county differences in distribution [70]. The community health arm has been boosted by establishment of community units and staffed with community health extension workers and community health volunteers [71]. The laboratory workers per 1000 population is low [53,71].	Workforce planning is fragmented, localised and not responsive to operational, geographical and population needs [72]. There are gaps in the health workforce including gender disparities in numbers and renumeration, with men occupying most senior role [72]. Only 3% of histopathology departments have sufficient staff to meet the clinical demand [73]. A big number of the available histopathologists are about to retire [74].
**Governance**	National level plans exist and cover activities important for pandemic/epidemic response *e.g.* disease surveillance and response [75]. There are national strategies for community engagement but with funding gaps [76]. There are disease specific community engagement strategies/guidelines [77]. There is a strong legislative arm (parliament) with a health committee that passes health related bills [77]. Parliament is involved in resource mobilisation for health including passing supplementary budgets for health *e.g.* during emergencies [78,79]. There are still concerns on data quality and completeness, and this affects decision making [80,81].	National level policies/plans/guidelines exist and align to global aspirations (UHC, SDGs, IHR *etc.*) [82,83]. Rules and guidelines are developed in response to health emergencies *e.g.* during COVID-19 [84]. Execution of rules and guidelines is high handed [85]. There are guidelines to streamline operations of private care providers but with weaknesses in enforcement and quality [86].	There is a long-term development plan and country specific plans made through stakeholder engagement including patient groups [32,87]. The country subscribes to the shared decision-making and documents to effect this exist [88-91]. Surveys show people don’t feel well empowered [92]. Parliament is able to pass laws during health emergencies through emergency legislation [93]. Parliament is able to pass supplementary budgets during health emergencies [62].
**Health information systems**	Absence of regulatory, financial frameworks *e.g.* absence of public funding for digital health, legal framework for data protection, protocols for regulating or certifying devices, frameworks for cross border data security and sharing [94]. There are limited cadres available for health information systems [95]. Reports from health facilities are often incomplete [96]. Several electronic systems for data capture are available but these are fragmented and lack integration [97,98]. Both paper and electronic data collection systems are in use including for laboratory data [99,100]. There are laboratory connectivity platforms to connect diagnostic technologies *e.g.* GxAlert, LabXpert [101,102].	There is a supportive policy environment for health information systems [103,104]. Several health information systems are in use such [105]. There is a lack of appropriate communication infrastructure and data security for DHIS2 that is widely used [106-108]. Internet connectivity challenges affect use of electronic data systems [109]. There are patients concerns about data use and privacy [110]. There is limited guidance on how to use the data [111]. Reporting of diagnostic and laboratory tests by general hospitals in the DHIS2 is minimal [74,112].	There are policy frameworks to support health information systems [113,114]. There are many electronic data systems but these are not interoperable [113]. There is evidence of data use to inform decision making *e.g.* to develop shielded lists to protect the vulnerable from COVID-19 [115]. Real time access to data are still a challenge [115].

DLSP – diagnostic and laboratory systems program, EMHS – essential medicines and health supplies

### Block 1: health service delivery

#### Number of laboratories

The decentralised nature and subsequent increase in the number of laboratories in Uganda, Kenya and the UK support increased access to laboratory services. The number of health facilities in Uganda and Kenya has gradually risen, hence improving health facility density [28,39]. Given that laboratories are established in these facilities, the increase in health facility density has led to increased access to testing by the population. In the UK, laboratory services underwent a redesign, which saw the consolidation of laboratory services into fewer hub laboratories while acute laboratory services were delivered in hospitals [116]. The hubs provide non-urgent specialist laboratory services to regional healthcare organisations or National Health Service (NHS) trusts, and allow laboratories to benefit from economies of scale, newer technologies, shared expertise and experience [117].

A weakness noted in Uganda is there are regional differences in density, with higher density in central Uganda than other regions [22], while in Kenya four out of every six health facilities are located in the capital (Nairobi) [118], and this could potentially affect surveillance, detection and reporting of public health pathogens of interest.

#### Access to laboratory services

There is a laboratory network in Uganda and Kenya, coordinated at the central level by centres of excellence (CoEs), also known as reference laboratories [21,29], that can do surveillance, early detection and control of epidemics. These include a national influenza centre and a virology laboratory, among others. Access to laboratory testing has been increased through community-level testing and the establishment of mobile laboratories [26,119]. Access to laboratory services has also been improved through the specimen referral system in Kenya and Uganda [32,119], and the pathology networks (hub and spoke) in the UK [120]. Under the hub and spoke model used in the UK, a central ‘hub’ laboratory provides non-urgent and specialist testing services for several regional healthcare organisations, and in cases where test results are needed in a very short time, smaller on-site essential services laboratories (the spokes) perform these tests [120]. Further, there is a vibrant private laboratory services sector in the three countries, with laboratories established within the private health facilities and stand-alone laboratories [31].

Weaknesses in Uganda and Kenya include the fact that the CoEs are located in the capital, and sub-optimal functionality of lower laboratories due to non-functioning equipment and a lack of equipment and reagents [24,25]. This could compromise the ability of laboratories to support an epidemic/pandemic response.

#### Multi-disease testing platforms

In Uganda and Kenya, molecular diagnostic technologies exist to support the diagnosis of pathogens of epidemic and pandemic potential. This has been effected through a massive roll-out of GeneXpert technology to support testing of tuberculosis (TB), HIV viral load, human papillomavirus, among others. This technology was repurposed for COVID-19 testing during the pandemic, and the same could be done for other infections of epidemic/pandemic potential. Kenya can be credited for the rapid uptake of the GeneXpert technology, introduced in Kenya in 2011, a year after being endorsed by WHO [121]. By 2018, there were 183 GeneXpert machines [122], which are anticipated to increase to 211 [123]. In Kenya, a diagnostic and laboratory systems program has developed and evaluated a TaqMan Array Card (TAC) technology, which can detect more than 25 pathogens from a single specimen [32].

There are challenges, *e.g.* GeneXpert coverage is low with in Uganda, with 227 machines in 227 of the 1500 (15%) of the TB diagnostic units available in the country [124]. This coverage is lower than the national target of 80%, and disparity in access has been noted, potentially curtailing response efforts. In Uganda and Kenya, challenges such as underutilisation of the GeneXpert machines exist [125].

### Block 2: health financing

#### Laboratory funding

Development partners supplement funding for the healthcare system in general and laboratory systems in particular, in Uganda and Kenya. Donor dependence in Kenya is exemplified by the American Centres for Disease Control (CDC)- funded diagnostic and laboratory systems program, which has improved laboratory systems for surveillance and outbreak investigation [32].

Donor dependence, however, is challenging as the funding is often not linked to government priorities. In Uganda, for example, the development partners’ support to the laboratory is most often related to vertical programs (*e.g.* HIV and TB), and only a few of the projects are linked to strengthening laboratories for diagnosis and surveillance [40]. In the UK, chronic underfunding and undervaluing of disciplines supporting infection testing, microbiology and virology have been reported [51]. A report of pathology services in England noted a decrease in NHS expenditure on pathology from 4% to 1.5–3% [126].

### Block 3: medical products, vaccines, and technologies

#### Spending

Laboratory commodities are crucial for diagnostics, surveillance, and disease control for epidemics/pandemics. In Uganda, funding for laboratory commodities has not been increased to match the increasing demand [127]. In the financial year (FY) 2020/2021, the government contribution to essential medicines health supplies (EMHS) in respect to the laboratory was only 9%, while the donor contribution was up to 91%. This could affect the laboratory response before and during a pandemic/epidemic.

In Kenya, there is an underfunding of health products and technologies (health products and technologies receive only 13% of the health budget) [52]. In the FY 2020/21, only 35% of the budget needed was availed, creating a funding gap of 65%, and laboratory supplies were allocated 6%, yet 24% of the budget would have been required [28].

In the UK, the NHS spends on average GBP 6 billion on hospital consumables, including gloves, syringes, and common goods [87]. However, it has been reported that the funding does not match the increased demand, and deficiencies have been noted, such as the supply chain issues noted during COVID-19 [51].

#### Availability of laboratory commodities

Stockouts could affect laboratory performance both before and during disease outbreaks. In Uganda, more than 90% of health centres and hospitals report stockouts of one or more tracer commodities across the system [128]. A basket of tracer commodities was available 81% of the time in 90 days in 198 health facilities, below the national target of 90% [39]. The availability of the laboratory tracer products was low at 78% compared to other baskets.

In Kenya, the average order fill rate for 14-tracer laboratory diagnostic supplies by quantity stands at 54%, with only half of the needed supplies being availed [28].

The UK struggles with insufficient stockpiles, and during the COVID-19 pandemic, it had to rely on supplies from China [51]. The UK has been criticised for not using the previous outbreaks (severe acute respiratory syndrome and Middle East respiratory syndrome outbreaks) to stockpile laboratory supplies that could have been used for the COVID-19 response.

### Block 4: human resources

#### Health worker training

In Uganda, reports show inequalities in student enrolment in medical training institutions. There are inequalities based on gender, geographical region, and the secondary schools attended. Females have less access than males, and the likelihood of joining a graduate institution is higher if the student attended a primary or secondary school in central Uganda [129]. Further, there are gaps in medical training, such as weak infrastructure, inadequate tutors, and frequent stockouts of the essential kits and equipment needed to train students [130].

Over the years, in Kenya, the number of medical laboratory graduates who applied for registration as a prerequisite for being added onto the national register has declined, which is attributed to stringent registration regulations [131]. There are gaps in the quality and adequacy of the curricula in the training institutions, with curricula not responsive to emerging issues in the health sector [132].

In the UK, the medical graduates per 100 000 inhabitants have increased from 11.05 in 2004 to 13.52 in 2022 [133]. Despite the increase, many people have never practiced in the UK. A survey among UK medical graduates showed that 23.52% intended to emigrate to practice medicine abroad, while 0.76% planned to leave the profession permanently [134]. Further, medical training costs are prohibitive and could affect the number of students enrolled. Training of healthcare professionals is expensive (GBP 66 000 for a registered nurse, 393 000 for a GP, and 516 000 for a consultant) [72].

The weaknesses above could affect the quantity and quality of personnel available to deliver laboratory services, as laboratory personnel are an integral aspect of a public health laboratory system and of attaining the IHR core capacities.

#### Laboratory personnel numbers

The laboratory professionals available per population in Uganda and Kenya are below the world average [53]. In Uganda, an absence of a sufficient number of laboratory technologists has been noted, with inequitable distribution across the levels of the healthcare system [67]. In Kenya, the number of laboratory personnel has improved over time. However, county differences in number and density exist [71]. The majority are in Nairobi; however, higher densities are noted in a few counties as well.

In the UK, only 3% of histopathology departments have sufficient staff to meet the clinical demand, and a big number of the available histopathologists are about to retire [73]. The NHS has struggled to recruit laboratory professionals for rural and remote areas [135]. The histopathology services are severely constrained, and the laboratories occasionally outsource work and use locums to fill these gaps [136].

### Block 5: governance

#### Legislation

In Uganda, the legislative arm (parliament) supports many health programs/initiatives, and in emergencies, the parliament supports resource allocation efforts, as was the case during COVID-19, when supplementary budgets were passed [78,79]. While the budgets were used to strengthen the overall response, some funds supported aspects of the laboratory system, such as tests, sample transport, and laboratory personnel allowances.

In Kenya, rules and executive orders were passed during COVID-19, *e.g.* the COVID-19 restriction of movement of persons and related measures of 2020 [84]. Whereas such orders were for the public good, in an epidemic/pandemic, they could potentially limit population movement to testing sites. In the presence of such orders, there should have been investments in bringing tests closer to the people, *e.g.* by providing test kits for self-testing, which was not available.

The UK parliament passed supplementary budgets to support the COVID-19 response [62]. Emergency legislation was adopted to contain the pandemic, and the new and old Acts became part of the response to the pandemic [137]. The ability to make legislation supporting pandemic response could be leveraged to enforce legislation in support of investments for laboratory strengthening, such as research and development to produce laboratory diagnostics and supplies.

#### Private sector role

In the three countries, the private sector supports the government in providing laboratory services, and such public-private partnerships could strengthen the IHR's laboratory core capacity.

In Uganda, the Ministry of Health approved private laboratories to perform COVID-19 testing [138]. In Kenya, the private sector represents 45% of the healthcare market [139], and 46% of the health facilities are private [140].

One challenge to leveraging the private sector during epidemics and pandemics is the cost of accessing private care. The exorbitant costs charged by private laboratories will likely hinder leveraging this sector for laboratory services during disease outbreaks. A study done in Kenya found that private in-facility laboratories charge an average test price of up to 468% compared to the average test in public laboratories [141]. In contrast, in Uganda, the cost of the COVID-19 tests was high, and experts mentioned private facilities were profiting off the pandemic [142]. In Uganda and Kenya, there are challenges with streamlining private sector services. Country-wide implementation of the policies is weak and poorly coordinated, with inadequate involvement and regulation of private providers [143].

In the UK, the contribution of private healthcare is on the rise as the population moves to private healthcare providers due to the frustration experienced in public health institutions [144]. The NHS also contracts out services to the private sector *e.g.* psychiatric and geriatric services [145], and the same could be done for providing laboratory services before and during epidemics/pandemics.

### Block 6: health information systems

#### Laboratory information systems

In Uganda, the national laboratory's primary data collection and reporting tools are paper-based, but there have been attempts at making them electronic [99]. Challenges in transitioning to the electronic laboratory information systems have been noted, including internet connectivity challenges, human resource gaps and hardware gaps [146], and this could affect the availability of laboratory systems to provide data to support the response to an epidemic or pandemic.

In Kenya, there is evidence of suboptimal use of laboratory information systems, for example, the reporting of diagnostic and laboratory tests by general hospitals in the DHIS2 Kenya is poor, with only 41.3% of the hospitals submitting monthly DHIS2 reports [74]. A survey among 219 health facilities revealed challenges with accessing the laboratory information management technology [112]. The laboratories in Uganda and Kenya have adopted connectivity solutions linked to some of the diagnostics currently in use for several diseases, such as the Gx Alert System connected to the GeneXpert machines [101,102].

#### Data use

In Uganda and Kenya, despite guidelines and efforts to improve the quality of data in the health information systems, there is limited guidance on how to use the data [111].

The UK demonstrated use of data and data systems during COVID-19 by producing shielded lists to protect vulnerable individuals from COVID-19 [115]. The country used databases such as hospital admissions, prescriptions and electronic medical records for primary care to develop algorithms to identify high-risk individuals. The biggest challenge was that the data was not accessible in real time.

## DISCUSSION

The review revealed findings across the three countries which could affect the laboratory, a core capacity of the IHR 2005. The core capacity requires member states to establish mechanisms for providing reliable and timely laboratory identification and characterisation of infectious agents, and other hazards likely to cause public health emergencies of national and international concern [101]. This could affect laboratory systems' ability to respond to current and future pandemics.

Regarding service delivery, the decentralised laboratory system in Uganda and Kenya enhances access. This attribute is critical in disease outbreaks as it enhances access to basic and sophisticated tests. Decentralisation of health services has been documented to improve access and increase hospital attendance [147]. In LMICs, there are several other benefits of decentralisation, such as motivation of local participation in health. In the LMICs, the local participation should be further enhanced through the provision of rapid diagnostic tests and subsequent empowerment of the population to do self-testing and interpret the results. In the high-income countries where this was done, it revolutionised COVID-19 testing [148]. Further, there is in-country capacity to diagnose hazardous pathogens as evidenced by the presence of centres of excellence (CoEs), also known as reference laboratories. The CoEs have capacity to do surveillance, early detection, and control of epidemics. However, in both Uganda and Kenya, these reference laboratories are situated in the capital cities, which could compromise accessibility during pandemics, and in the presence of a poor road network, this leads to delayed testing and reduced quality of specimens [149]. Policies should be instituted to support establishing and accreditation regional reference laboratories. These should be equipped with sufficient capacity, biosecurity measures and enrolled into external quality assessment schemes for major public health disciplines.

Specimen collection, handling, packaging and transportation are crucial for accurate diagnosis and effective health responses per the IHR framework. The review revealed this is affected through specimen referral systems, and the hub and spoke system. A sound specimen referral system has the potential to improve global health security through effective disease control and prevention [150]. The hub and spoke system has been found to lower the cost of tests, lead to savings from joint procurements, alleviate staff shortages and reduce delays to diagnostic results [37,151]. The specimen referral systems however still face challenges *e.g.* the hub and spoke system has been blamed for creating redundancy among some laboratories and creating delays as the laboratories are located at sites distant from where the services can be accessed [51]. Similar challenges exist in Uganda and Kenya, and strategies to overcome these challenges should be sought, *e.g.* by developing a training curriculum, training, mapping of facilities through GPS and GIS technology, provision of supplies and public private partnerships [152].

Integral to the IHR framework is the availability of and timely access to reliable information. The review noted that laboratories in the study countries have embraced the connectivity solutions linked to some of the diagnostics currently in use for several diseases, such as the Gx Alert system that is connected to the GeneXpert machines [101,102]. These solutions help in decision making by providing timely data, which is key for diagnostic technology utilisation rates [102], and results dissemination to support prompt clinical decision-making during outbreaks. These connectivity solutions support data driven strategies such as modelling during pandemics which rely on real data stream to monitor the pandemic, model and forecast the pandemic and manage the pandemic [153], which could also potentially contribute to surveillance, which is a core capacity of the IHR 2005 by supporting access to real time data and hence timely reporting and enhancement of early warning systems. There are challenges with transitioning to laboratory information systems, including limited internet connectivity and human resource and hardware gaps [146]. To overcome these, countries should ensure a supportive environment for digital health and health information technology, *e.g.* by developing legal frameworks for data protection, including digital health in health and related professional in-service training. Digital health plans should be well-costed and funded, as funding has been identified as one of the ingredients to support implementation of digital plans [154]. In the LMICs, the decentralisation of community-level testing through rapid diagnostic tests should be supported by investments in recording and reporting systems at this level to inform a response during and before disease outbreak. The community health workers or village health teams that are part of the community health system in most LMICs could be empowered to do digital recording and reporting *e.g.* through mobile applications. Standards should be established to allow the creation of systems in which data can be readily shared and used across platforms.

A well-stocked laboratory supports prevention, detection, control and rapid response to public health threats, a goal of the IHR. The review revealed suboptimal functionality of the laboratories due to issues such as stock out of supplies, and equipment breakdown. The stockouts get more exacerbated by increased global demand and shipping delays during pandemics [155]. There is a need to make laboratories more functional to be better prepared for current and future outbreaks. In LMICs, investments in local engineering solutions, including biomedical engineers, could be adopted [156].

Laboratory personnel are an integral aspect of a public health laboratory system, and attainment of the IHR core capacities is hinged on staff availability in sufficient numbers with the required training. The review revealed low numbers of laboratory and histopathology personnel in the study countries. The laboratory professionals per 1000 population in Kenya and Uganda is lower than the world average [53], while in the UK only 3% of histopathology departments have sufficient numbers, with majority about to retire [73]. Countries should explore avenues to increase workforce levels through interventions such as the development of early career apprenticeship programs to be used as an entry route to the laboratory science profession, the development of approaches for raising the profile of life sciences and the career opportunities within training institutions such as colleges and universities [135]. Changes in workforce through use of skills mix initiatives and the establishment of new roles and hybrid teams have potential to respond to laboratory workforce shortages [136].

The private health sector delivers a significant proportion of healthcare [157]. During COVID-19, the private health sector was instrumental in supporting the government-level efforts in increasing COVID-19 testing in both LMICs and HICs [16,122]. Countries should explore ways of working collaboratively with this sector while ensuring they are held accountable to high standards of care delivery [157]. Policies to strengthen the performance of this sector should be developed, such as tax incentives, direct government support to private healthcare providers through funding, health worker training, provision of vouchers to be used in private facilities, strengthening and streamlining frameworks, provision of drugs, and contracting out services to private providers [158,159].

### Limitations

This review included only literature in English, as it was difficult to review literature in languages other than English. However, English is primarily used in the study countries, and it is thus possible that not a lot of literature was missed. Another limitation is that for each of the building blocks, it was not possible to do a country comparative review due to the unavailability of sufficient data from the literature accessed. Thus, the review was done to the extent it was made possible by the available literature. A third limitation is that the review relied on secondary sources, which could be inaccurate and introduce bias.

## CONCLUSIONS

This review revealed that all countries possess strengths that could be consolidated and weaknesses that should be addressed as part of pandemic preparedness. The growing contribution of the private sector in the three countries and deployment of multi-disease testing platforms should be consolidated, given the advantage of shared financial costs in the face of chronic underfunding for the laboratory systems and healthcare in general.

## References

[1] World Health Organization. Statement on the fifteenth meeting of the IHR 2005 Emergency Committee on the COVID-19 pandemic. 5 May 2023. Available: https://www.who.int/news/item/05-05-2023-statement-on-the-fifteenth-meeting-of-the-international-health-regulations-(2005)-emergency-committee-regarding-the-coronavirus-disease-(covid-19)-pandemic?adgroupsurvey=%7Badgroupsurvey%7D&gclid=CjwKCAjwxOymBhAFEiwAnodBLLN0q2GjrWDafgxoOOah69OiWvyEtY9uCYaOH5Plj8BOj4b0lxcvhxoCw-QQAvD_BwE. Accessed: 15 August 2023.

[2] World Health Organization. WHO COVID-19 dashboard. 2020. https://covid19.who.int/?adgroupsurvey=%7Badgroupsurvey%7D&gclid=CjwKCAjwxOymBhAFEiwAnodBLFLyB6ltLhD2oDC1oNC8iT6JT7g6Z_ngkpWnj0v90exAqqPGXtPBVRoCIpEQAvD_BwE. Accessed: 15 August 2023.

[3] Liu Y Morgenstern C Kelly J Lowe R The impact of non-pharmaceutical interventions on SARS-CoV-2 transmission across 130 countries and territories. BMC Med. 2021;19:40. 10.1186/s12916-020-01872-8 33541353 PMC7861967

[4] Rowan NJ Moral RA Disposable face masks and reusable face coverings as non-pharmaceutical interventions (NPIs) to prevent transmission of SARS-CoV-2 variants that cause coronavirus disease (COVID-19): Role of new sustainable NPI design innovations and predictive mathematic. Sci Total Environ. 2021;772:145530. 10.1016/j.scitotenv.2021.145530 33581526 PMC7848491

[5] Chakraborty C Sharma AR Bhattacharya M Agoramoorthy G Lee SS The Drug Repurposing for COVID-19 Clinical Trials Provide Very Effective Therapeutic Combinations: Lessons Learned From Major Clinical Studies. Front Pharmacol. 2021;12:704205. 10.3389/fphar.2021.704205 34867318 PMC8636940

[6] Wen W Chen C Tang J Wang C Zhou M Cheng Y Annals of Medicine Efficacy and safety of three new oral antiviral treatment (molnupiravir, fluvoxamine and Paxlovid) for COVID-19: a meta-analysis. Ann Med. 2022;54:516–23. 10.1080/07853890.2022.2034936 35118917 PMC8820829

[7] Ioannidis JPA Pezzullo AM Cristiano A Boccia S Global estimates of lives and life-years saved by COVID-19 vaccination during 2020-2024. medRxiv: 24316673v1. 2024. Available: https://www.medrxiv.org/content/10.1101/2024.11.03.24316673v1. Accessed: 19 June 2025. 10.1101/2024.11.03.24316673 40711778

[8] Lippi G Plebani M The critical role of laboratory medicine during coronavirus disease 2019 (COVID-19) and other viral outbreaks. Clin Chem Lab Med. 2020;58:1063–9. 10.1515/cclm-2020-0240 32191623

[9] Bohn MK Lippi G Horvath A Sethi S Koch D Ferrari M Molecular, serological, and biochemical diagnosis and monitoring of COVID-19: IFCC taskforce evaluation of the latest evidence. Clin Chem Lab Med. 2020;58:1037–52. 10.1515/cclm-2020-0722 32459192

[10] Ondoa P Kebede Y Loembe MM Bhiman JN Tessema SK Sow A COVID-19 testing in Africa: lessons learnt. Lancet Microbe. 2020;1:e103–4. 10.1016/S2666-5247(20)30068-9 32835338 PMC7333988

[11] Badu K Thorn JPR Goonoo N Dukhi N Fagbamigbe AF Kulohoma BW Africa ’ s response to the COVID-19 pandemic: A review of the nature of the virus, impacts and implications for. AAS Open Res. 2020;3:19. 10.12688/aasopenres.13060.1

[12] Lau H Khosrawipour T Kocbach P Ichii H Bania J Khosrawipour V Evaluating the massive underreporting and undertesting of COVID-19 cases in multiple global epicenters. Pulmonology. 2021;27:110–5. 10.1016/j.pulmoe.2020.05.015 32540223 PMC7275155

[13] Wolford T Sutton J Mangal CN Laboratory Response to Pandemic Threats: Challenges, Needs, and Solutions. Health Secur. 2023;21:S56–9. 10.1089/hs.2022.0123 37565776 PMC10818051

[14] Devkota JU Estimation of Underreported Cases of Infections and Deaths from COVID-19 for Countries with Limited and Scarce Data: Examples from Nepal. J Environ Public Health. 2022;2022:3276583. 10.1155/2022/3276583 35024047 PMC8743622

[15] Kobia F Gitaka J COVID-19: Are Africa’s diagnostic challenges blunting response effectiveness? AAS Open Res. 2020;3:4. 10.12688/aasopenres.13061.1 32399515 PMC7205356

[16] Kebede A Lanyero B Beyene B Mandalia ML Melese D Girmachew F Expanding molecular diagnostic capacity for COVID-19 in Ethiopia: operational implications, challenges and lessons learnt. Pan Afr Med J. 2021;38:68. 33889234 10.11604/pamj.2021.38.68.27501 PMC8028356

[17] Armani AM Diebold ED Catalyzing pathways for translational research beyond COVID-19. Commun Phys. 2021;4:167. 10.1038/s42005-021-00672-7

[18] Alexander M Unruh L Koval A Belanger W United States response to the COVID-19 pandemic, January–November 2020. Health Econ Policy Law. 2022;17:62–75. 10.1017/S1744133121000116 33663642 PMC8060542

[19] United States Agency for International Development. Health Systems Assessment Approach: A How-to Manual. Washington D.C., USA: United States Agency for International Development; 2007. Available: https://cdi.mecon.gob.ar/bases/doc/phr/publicaciones/1.pdf. Accessed: 19 June 2025.

[20] Popay J, Roberts H, Sowden A, Petticrew K, Arai L, Rodgers M, et al. Guidance on the Conduct of Narrative Synthesis in Systematic Reviews – A Product from the ESRC Methods Programme. Lancaster, UK: Lancaster University; 2006. Available: https://www.researchgate.net/publication/233866356_Guidance_on_the_conduct_of_narrative_synthesis_in_systematic_reviews_A_product_from_the_ESRC_Methods_Programme. Accessed: 8 July 2025.

[21] Ministry of Health of Uganda. MINISTRY OF HEALTH STRATEGIC PLAN 2020/21 - 2024/25. Kampala, Uganda: Ministry of Health of Uganda; 2021. Available: https://extranet.who.int/countryplanningcycles/sites/default/files/public_file_rep/UGA_Uganda_Ministry-of-Health-Strategic-Plan_2020-2025.pdf. Accessed: 19 June 2025.

[22] Ministry of Health of Uganda. Uganda Hospital and Health Centre IV Census Survey. Kampala, Uganda: Ministry of Health of Uganda; 2014. Available: https://health.go.ug/sites/default/files/Hospital%20Census%20Report%20Jan%202016.pdf. Accessed: 19 June 2025.

[23] Uganda National Health Laboratory Services. Laboratory Services | Central Public Health Laboratories. 2025. Available: https://cphl.go.ug/home-01. Accessed: 17 August 2023.

[24] Namuhani N Kiwanuka SN Akulume M Kalyesubula S Bazeyo W Kisakye AN Laboratory Diagnostics Performance in Uganda: A Survey of Test Availability and Constraints Across 100 Laboratories. J Interval Epidemiol Public Health. 2024;7:10.

[25] Matovu M Musiime E Olak P Mulindwa M Namisango E Songwe K Impact of accreditation on health care services performance in Kiryandongo district, Uganda: a longitudinal study. BMC Health Serv Res. 2022;22:174. 10.1186/s12913-022-07603-4 35144593 PMC8830999

[26] Ministry of Health of Uganda. National Tuberculosis and Leprosy Revised National Strategic Plan 2015 /16-2019/20. Kampala, Uganda: Ministry of Health of Uganda; 2017. Available: https://health.go.ug/sites/default/files/Revised%20NTLP-Stragetic%20Plan%20%282015-2020%29_25th%20Jul2017%20final.pdf. Accessed: 19 June 2025.

[27] Central Public Health LaboratoriesHome. 2025. Available: https://www.cphl.go.ug/. Accessed: 9 July 2025.

[28] Ministry of Health of Kenya. Health Sector Annual Report. Nairobi, Kenya: Ministry of Health of Kenya; 2014. Available: https://healthgarissa.go.ke/wp-content/uploads/2024/09/HIS-Annual-Report-2014_Final.pdf. Accessed: 19 June 2025.

[29] Ministry of Health of Kenya. National Public Health Laboratories – Efficient, Accessible Responsive and High Quality Public Health Laboratory. 2025. Available: https://nphl.go.ke/. Accessed: 14 March 2023.

[30] Kenya National Public Health Insititute. National Virology Reference Laboratory – National Public Health Laboratories. 2025. Available: https://www.nphi.go.ke/national-virology-reference-lab. Accessed: 14 March 2023.

[31] Ministry of Health of UgandaHome. 2019. Available: www.health.go.ug. Accessed: 19 April 2021.

[32] Hunsperger E Juma B Onyango C Ochieng JB Omballa V Fields BS Building laboratory capacity to detect and characterize pathogens of public and global health security concern in Kenya. BMC Public Health. 2019;19:477. 10.1186/s12889-019-6770-9 32326916 PMC6696698

[33] Ministry of Health of Kenya. Kenya Laboratory Capacity Mapping Report. Nairobi, Kenya: Ministry of Health of Kenya; 2020. Available: https://www.nphi.go.ke/sites/default/files/2024-02/Kenya-Laboratory-Capacity-Mapping-Report-February-2020-compressed.pdf. Accessed: 21 June 2025.

[34] Lehmann J, Muture B, Matu M, Schneidman M. Performance Evaluation of Public Health Laboratories in Kenya. New Hampshire, USA: World Bank; 2018. Available: https://www.researchgate.net/publication/326200926_Performance_Evaluation_of_Public_Health_Laboratories_in_Kenya. Accessed: 21 June 2025.

[35] Exeter Clinical Laboratory International. National Pathology Exchange (NPEx). 2022. Available: https://www.exeterlaboratory.com/national-pathology-exchange-npex/. Accessed: 19 May 2023.

[36] United Kingdom Accreditation ServiceAbout us. 2015. Available: https://www.ukas.com/about-us/. Accessed: 6 June 2023.

[37] National Health Service England. NHS Improvement Operational Productivity Proposed Pathology Consolidation Networks. London, UK: National Health Service England; 2017. Available: https://www.england.nhs.uk/wp-content/uploads/2021/05/proposed-pathology-consolidation-networks.pdf. Accessed: 19 May 2023.

[38] National Health Service. Pathology quality assurance dashboard: second edition. London, UK: National Health Service; 2019. Available: https://www.england.nhs.uk/wp-content/uploads/2020/08/Pathology_quality_assurance_dashboard_PQAD.pdf. Accessed: 21 June 2025.

[39] Ministry of Health of Uganda. Annual Health Sector Performance Report 2020-21. Kampala, Uganda: Ministry of Health of Uganda; 2021. Available: https://library.health.go.ug/sites/default/files/resources/Annual%20Health%20Sector%20Performance%20Report%202020-21-1.pdf. Accessed: 19 June 2025.

[40] Ministry of Health of Uganda. Tracking off budget financial resources in the health sector FY2018-19. Kampala, Uganda: Ministry of Health of Uganda; 2020. Available: https://www.unicef.org/uganda/media/7276/file/Tracking%20off%20budget%20financial%20resources%20in%20the%20health%20sector%20FY2018-19-lores.pdf. Accessed: 19 June 2025.

[41] The World Bank. Some Days Are Better Than Others: Lessons Learned from Uganda’s First Results-Based Financing Pilot. New Hampshire, USA: World Bank; 2007. Available: https://documents1.worldbank.org/curated/en/177451468121506594/pdf/539850BRI0RBF110Box345633B01PUBLIC1.pdf. Accessed: 21 June 2025.

[42] Open Enabel. Roll out the National Results Based Financing Policy in the Acholi Sub-Region, Uganda (USAID EHA). 2022. Available: https://open.enabel.be/fr/UGA/2315/p/roll-out-the-national-results-based-financing-policy-in-the-acholi-sub-region-uganda-usaid-eha.html. Accessed: 14 March 2023.

[43] Living Goods. Results-Based Financing for Community Health: An innovative, results-oriented approach to ensuring accountability, driving cost-effectiveness, and delivering impact for donors and governments. 2018. Available: https://livinggoods.org/wp-content/uploads/2019/06/Results-Based-Financing-for-Community-Health_A4Size.pdf. Accessed: 26 August 2025.

[44] Ministry of Health of Kenya. National and County Health Budget Analysis FY 2020/21. Nairobi, Kenya: Ministry of Health of Kenya; 2022. Available: http://guidelines.health.go.ke:8000/media/National_and_County_Budget_Analysis_FY_2020-21_April_2022.pdf. Accessed: 26 August 2025.

[45] Kenya National Bureau of Statistics. Kenya Demographic and Health Survey 2022 Key Indicators Report. Nairobi, Kenya: Kenya National Bureau of Statistics; 2023. Available: www.DHSprogram.com. Accessed: 25 June 2025.

[46] Moses MW Korir J Zeng W Musiega A Oyasi J Lu R Performance assessment of the county healthcare systems in Kenya: a mixed-methods analysis. BMJ Glob Health. 2021;6:e004707. 10.1136/bmjgh-2020-004707 34167962 PMC8230973

[47] Global Burden of Disease 2021 Health Financing Collaborator Network Global investments in pandemic preparedness and COVID-19: development assistance and domestic spending on health between 1990 and 2026. Lancet Glob Health. 2023;11:e385–413. 10.1016/S2214-109X(23)00007-4 36706770 PMC9998276

[48] World Bank Group. Current health expenditure (% of GDP) - United Kingdom. 2019. Available: https://data.worldbank.org/indicator/SH.XPD.CHEX.GD.ZS?locations=GB. Accessed: 18 August 2023.

[49] Office for Budget Responsibility. Economic and fiscal outlook - October 2024. 2024. Available: https://obr.uk/efo/economic-and-fiscal-outlook-october-2024/. Accessed: 21 June 2025.

[50] Anderson M Pitchforth E Asaria M Brayne C Casadei B Charlesworth A LSE–Lancet Commission on the future of the NHS: re-laying the foundations for an equitable and efficient health and care service after COVID-19. Lancet. 2021;397:1915–78. 10.1016/S0140-6736(21)00232-4 33965070

[51] Banatvala J COVID-19 testing delays and pathology services in the UK. Lancet. 2020;395:1831. 10.1016/S0140-6736(20)31037-0 32473100 PMC7255215

[52] Ministry of Health Republic of Uganda. Roadmap for Government of Uganda’s Health Supply Chain Self-Reliance. 2020. Available: https://library.health.go.ug/medical-products-technologies/medicines/10-year-roadmap-government-ugandas-health-supply-chain-self. Accessed: 19 June 2025

[53] Japan International Cooperation Agency. BUILDING RESILIENCE COVID-19 IMPACT & RESPONSE IN URBAN AREAS - CASE OF KENYA & UGANDA. Tokyo, Japan: Japan International Cooperation Agency; 2020. Available: https://www.jica.go.jp/Resource/activities/issues/urban/ku57pq000019fbsv-att/kenya_uganda_02en_01.pdf. Accessed: 19 June 2025.

[54] Chen J Ssennyonjo A Wabwire-Mangen F Kim JH Bell G Hirschhorn L Does decentralization of health systems translate into decentralization of authority? A decision space analysis of Ugandan healthcare facilities. Health Policy Plan. 2021;36:1408–17. 10.1093/heapol/czab074 34165146 PMC8505862

[55] The Global Fund. Audit Report: Global Fund Grants in the Republic of Uganda. Geneva, Switzerland: The Global Fund; 2019. Available: https://www.theglobalfund.org/media/8804/oig_gf-oig-19-017_report_en.pdf. Accessed: 21 June 2025.

[56] Ministry of Health of Uganda. Essential Medicines and Health Supplies List for Uganda (EMHSLU). Kampala, Uganda: Ministry of Health of Uganda; 2016. Available: https://www.health.go.ug/wp-content/uploads/2019/11/Essential-Medicines-and-Health-Supplies-List_EMHSLU_2016_FINAL_0.pdf. Accessed: 21 June 2025.

[57] Ministry of Health of Uganda. National Standard Test Menu, Techniques and Supplies List for Laboratories. Kampala, Uganda: Ministry of Health of Uganda; 2017. Available: https://cphl.go.ug/web/sites/default/files/2024-10/NSTMT%20TEST%20MENU.pdf. Accessed: 21 June 2025.

[58] World Bank Blogs. Kenya’s medical supply agency transforms to improve service delivery and save lives. 31 August 2015. Available: https://blogs.worldbank.org/nasikiliza/kenyas-medical-supply-agency-transforms-to-improve-service-delivery-and-save-lives. Accessed: 1 August 2023.

[59] Kenya Medical Supplies Authority. Business model. 2018. Available: https://kemsa.go.ke/about-us. Accessed: 1 August 2023.

[60] National Taxpayers Association. Emergency Public Procurement in Kenya: The case of the health sector procuring for COVID-19. Nairobi, Kenya: National Taxpayers Association; 2020. Available: https://www.nta.or.ke/wp-content/uploads/2024/04/Emergency-Public-Procurement-in-Kenya.pdf. Accessed: 21 June 2025.

[61] Republic of Kenya, Ministry of Health. Kenya Essential Medical Medicines List 2019. Nairobi, Kenya: Ministry of Health; 2019. Available: https://khf.co.ke/wp-content/uploads/2020/03/KEML-2019.pdf. Accessed: 18 May 2023.

[62] Welsh Government. Supplementary Budget 2021-2022. Cardiff, Wales: Welsh Government; 2022. Available: https://www.gov.wales/sites/default/files/publications/2022-02/2nd-supplementary-budget-2021-2022-note.pdf. Accessed: 19 June 2025.

[63] National Audit Office. Investigation into government procurement during the COVID-19 pandemic. 26 November 2020. Available: https://www.nao.org.uk/reports/government-procurement-during-the-covid-19-pandemic/. Accessed: 21 June 2025.

[64] Ministry of Education & Sports. Health Education and Training. 2017. Available: https://www.education.go.ug/health-education-and-training/. Accessed: 18 May 2023.

[65] Monitor. EAC probe queries medical training in Ugandan varsities. 16 January 2021. Available: https://www.monitor.co.ug/uganda/news/national/eac-probe-queries-medical-training-in-ugandan-varsities-1654646. Accessed: 18 May 2023.

[66] Ministry of Health of Uganda. Human resources for health audit report. Kampala, Uganda: Ministry of Health of Uganda; 2018. Available: https://library.health.go.ug/sites/default/files/resources/Human%20Resources%20for%20Health%20Audit%20Report%202017-18.pdf. Accessed: 21 June 2025.

[67] Kiwanuka SN Namuhani N Akulume M Kalyesubula S Bazeyo W Kisakye AN Uganda’s laboratory human resource in the era of global health initiatives: Experiences, constraints and opportunities - An assessment of 100 facilities. Hum Resour Health. 2020;18:13. 10.1186/s12960-020-0454-5 32070361 PMC7029471

[68] Ministry of Public Service. Circular Standing Instruction No. 9 of 2018, Scheme of Service for Medical Laboratory Cadre in the Uganda Public Service. Kampala, Uganda: Ministry of Public Service; 2019. Available: https://www.cphl.go.ug/web/sites/default/files/2024-10/Schemes%20of%20service%20for%20Medical%20Laboratory%20Cadre%20January%202019%20%28SMK%29.docx%202.pdf. Accessed: 21 June 2025.

[69] Ministry of Health. Health CS inaugurates NHIF, KHPOA and PBB boards, gives assurance over planned healthcare reforms. Available: https://healthbusiness.co.ke/6350/kagwe-inaugurates-nhfi-khpoa-and-pbb-boards/. Accessed: 15 March 2023.

[70] Open AFRICA. Health workers in Kenya. 2020. Available: https://open.africa/dataset/health-workers-in-kenya. Accessed: 18 May 2023.

[71] Ministry of Health of Kenya. Health Sector Performance Report FY 2018/2019. Nairobi, Kenya: Ministry of Health of Kenya; 2018. Available

[72] Anderson M O’Neill C Macleod Clark J Street A Woods M Johnston-Webber C Securing a sustainable and fit-for-purpose UK health and care workforce. Lancet. 2021;397:1992–2011. 10.1016/S0140-6736(21)00231-2 33965066 PMC9634455

[73] The Royal College of Pathologists. Meeting pathology demand: Histopathology workforce census. London, UK: The Royal College of Pathologists; 2018. Available: https://www.rcpath.org/uploads/assets/952a934d-2ec3-48c9-a8e6e00fcdca700f/Meeting-Pathology-Demand-Histopathology-Workforce-Census-2018.pdf. Accessed: 19 June 2025.

[74] Bahati F Mcknight J Swaleh F Malaba R Karimi L Ramadhan M Reporting of diagnostic and laboratory tests by general hospitals as an indication of access to diagnostic laboratory services in Kenya. PLoS One. 2022;17:e0266667. 10.1371/journal.pone.0266667 35395040 PMC8992978

[75] National Planning Authority of Uganda. Third National Development Plan (NDP III) 2020/21-2024/25. Kampala, Uganda: National Planning Authority of Uganda; 2020. Available: https://www.npa.go.ug/wp-content/uploads/2023/03/NDPIII-Finale_Compressed.pdf. Accessed: 21 June 2025.

[76] Ministry of Health of Uganda. National Community Engagement Strategy for COVID-19 Response. Kampala, Uganda: Ministry of Health of Uganda; 2020. Available: https://static1.squarespace.com/static/5e7b914b3b5f9a42199b3337/t/5fde38047185ee572d414bd2/1608398854507/NATIONAL+COMMUNITY+ENGAGEMENT+STARTEGY+FOR+COVID-19+Book.pdf. Accessed: 21 June 2025.

[77] Parliament Watch. Committee on Health. 2021. Available: https://parliamentwatch.ug/committees/committee-on-health/. Accessed: 2 August 2023.

[78] Anti-Corruption Resource Centre. Uganda’s Covid-19 supplementary budget pandemic response or cash bonanza? 2020. Available: https://www.u4.no/blog/ugandas-covid-19-supplementary-budget-pandemic-response-or-cash-bonanza. Accessed: 2 August 2023.

[79] Watchdog News. Parliament passes Shs3.8 trillion supplementary expenditure. 2021. Available: https://www.watchdoguganda.com/news/20211120/125193/parliament-passes-shs3-8-trillion-supplementary-expenditure.html. Accessed: 2 August 2023.

[80] Ward K Mugenyi K Benke A Luzze H Kyozira C Immaculate A Enhancing workforce capacity to improve vaccination data quality, Uganda. Emerg Infect Dis. 2017;23:S85–93. 10.3201/eid2313.170627 29155675 PMC5711317

[81] Ronveaux O Rickert D Hadler S Groom H Lloyd J Bchir A The immunization data quality audit: verifying the quality and consistency of immunization monitoring systems. Bull World Health Organ. 2005;83:503–10. 16175824 PMC2626295

[82] Laws of Kenya. Public Health Act Chapter 242. Nairobi, Kenya: National Council for Law Reporting; 2012. Available: https://kenyalaw.org/kl/fileadmin/pdfdownloads/Acts/PublicHealthActCap242.pdf. Accessed: 21 June 2025.

[83] Republic of Kenya. Kenya Gazette Supplement: Kenya Health Act, 2017. Nairobi, Kenya: Republic of Kenya; 2017. Available: https://kenyalaw.org/kl/fileadmin/pdfdownloads/Acts/HealthActNo.21of2017.pdf. Accessed: 21 June 2025.

[84] Legal and Corporate Board Services University of Nairobi. Government legislation passed in tackling the COVID – 19 pandemic. 26 June 2020. Available: https://legaloffice.uonbi.ac.ke/latest-news/government-legislation-passed-tackling-covid-19-pandemic. Accessed: 3 August 2023.

[85] Sifuna N Mogere S Enforcing Public Health Law in Africa: Challenges and Opportunities, the Case of Kenya. Zambia Law J. 2002;34:148–59.

[86] The World Bank. Private Health Sector Assessment in Kenya. Int J Res Manag Econ Commer. New Hampshire, USA: The World Bank; 2010. Available: https://documents1.worldbank.org/curated/en/434701468048274776/pdf/552020PUB0Heal10Box349442B01PUBLIC1.pdf. Accessed: 21 June 2025.

[87] National Health Service. Facing the Facts, Shaping the Future. London, UK: National Health Service; 2017. Available: https://www.hee.nhs.uk/sites/default/files/documents/Facing%20the%20Facts,%20Shaping%20the%20Future%20%E2%80%93%20a%20draft%20health%20and%20care%20workforce%20strategy%20for%20England%20to%202027.pdf. Accessed: 21 June 2025.

[88] Kapur N The NHS Long Term Plan. Sushruta Journal of Health Policy & Opinion. 2020;12:10–11. 10.38192/12.1.4

[89] Coulter A Collins A Edwards A Entwistle V Finnikin S Joseph-Williams N Implementing shared decision-making in UK: Progress 2017–2022. Z Evid Fortbild Qual Gesundhwes. 2022;171:139–43. 10.1016/j.zefq.2022.04.024 35610131

[90] Coulter A Edwards A Elwyn G Thomson R Implementing shared decision making in the UK. Z Evid Fortbild Qual Gesundhwes. 2011;105:300–4. 10.1016/j.zefq.2011.04.014 21620325

[91] National Health Service England. Working in partnership with people and communities: Statutory guidance. 2022. Available: https://www.england.nhs.uk/long-read/working-in-partnership-with-people-and-communities-statutory-guidance/. Accessed: 21 June 2025.

[92] GOV.UK. Civic Engagement and Social Action - Community Life Survey 2020/21. 29 July 2021. Available: https://www.gov.uk/government/statistics/community-life-survey-202021-civic-engagement-and-social-action/civic-engagement-and-social-action-community-life-survey-202021. Accessed: 3 August 2023.

[93] Joint Committee on Human Rights. The Government’s response to Covid-19: human rights implications. London, UK: Joint Committee on Human Rights; 2020. Available: https://committees.parliament.uk/publications/2649/documents/26914/default/. Accessed: 21 June 2025.

[94] Global Digital Health Monitor. Global Digital Health Index. 2023. http://index.digitalhealthindex.org/country_profile/UGA. Accessed: 19 May 2023.

[95] Ministry of Health of Uganda. The Uganda Health Information and Digital Health Strategic Plan 2020/21-2024/25. Kampala, Uganda: Ministry of Health of Uganda; 2021. Available: https://library.health.go.ug/health-information-systems/digital-health/uganda-health-information-and-digital-health-strategic. Accessed: 21 June 2025.

[96] Alunyu AE, Wamema J, Kiwanuka A, Moses B, Amiyo M, Kambugu A, et al. Investigating the Impediments to Accessing Reliable, Timely and Integrated Electronic Patient Data in Healthcare Sites in Uganda. In: Gehin C, Wacogne B, Douplik A, Lorenz R, Bracken B, Pesquita C, Fred A, Gamboa H, editors. BIOSTEC 2021: Proceedings of the 14th International Joint Conference on Biomedical Engineering Systems and Technologies; 2021 Feb 11–13; Lyon, France. California, USA: Curran and Associates Inc.; 2021. p. 522–532.

[97] Ministry of Health of Uganda. Strengthening Uganda’s Health System through Standardising digital health. Kampala, Uganda: Ministry of Health of Uganda; 2021. Available: https://library.health.go.ug/health-information-systems/digital-health/strengthening-ugandas-health-system-through-standardizing. Accessed: 21 June 2025.

[98] Huang F Blaschke S Lucas H Beyond pilotitis: Taking digital health interventions to the national level in China and Uganda. Global Health. 2017;13:49. 10.1186/s12992-017-0275-z 28756767 PMC5535287

[99] Ministry of Health of Uganda. Uganda National Health Laboratory Services Policy II. Kampala, Uganda: Ministry of Health of Uganda; 2016. Available: https://cphl.go.ug/web/sites/default/files/2024-10/UG%20NHLS%20Policy%20%20LTC%20Final%20draft%20-%20Thomas-Pizaro-Gaspard%20signed23Mar2018.pdf. Accessed: 19 June 2025.

[100] Alunyu AE, Wamema J, Kiwanuka A, Moses B, Amiyo M, Kambugu A, et al. Investigating the Impediments to Accessing Reliable, Timely and Integrated Electronic Patient Data in Healthcare Sites in Uganda. In: Gehin C, Wacogne B, Douplik A, Lorenz R, Bracken B, Pesquita C, Fred A, Gamboa H, editors. BIOSTEC 2021: Proceedings of the 14th International Joint Conference on Biomedical Engineering Systems and Technologies; 2021 Feb 11–13; Lyon, France. California, USA: Curran and Associates Inc.; 2021. p. 522–532.

[101] National Tuberculosis and Leprosy Program Ministry of Health. Country experiences with optimizing TB diagnostic and sample transport networks to improve access to rapid testing: Uganda. African Regional TB Summit post- UNHLM: Step up effort to find all people with TB. 2025.

[102] Stop TB Partnership. Diagnostics Connectivity Solution Profile: GxAlert / Aspect. 2021. Available: https://www.stoptb.org/sites/default/files/imported/document/gxalert.pdf. Accessed: 26 August 2025.

[103] The National Treasury and Planning. THIRD-MEDIUM-TERM-PLAN-2018-2022. Nairobi, Kenya: The National Treasury and Planning; 2018. Available: https://vision2030.go.ke/wp-content/uploads/2019/01/THIRD-MEDIUM-TERM-PLAN-2018-2022.pdf. Accessed: 21 June 2025.

[104] World Health Organization. Kenya National e-Health Strategy 2011-2017. 2011. Available: https://extranet.who.int/mindbank/item/1956. Accessed: 21 June 2025.

[105] Ministry of Health of Kenya. Kenya Health Sector Strategic Plan 2018-2023 Mid Term Review Synthesis Report. Nairobi, Kenya: Ministry of Health of Kenya; 2021. Available: https://uniatf.who.int/docs/librariesprovider22/default-document-library/khssp-mtr-statistical-report-march-2021.pdf?sfvrsn=f6ba673f_1. Accessed: 21 June 2025.

[106] Palladium. Kenya Health Information Systems. 2022. Available: https://kenyahmis.org/about/. Accessed: 15 March 2023.

[107] United Nations Children’s Fund. ASSESSING PARTNER ALIGNMENT IN SUPPORT OF THE HEALTH INFORMATION SYSTEM IN KENYA. New York, USA: United Nations Children’s Fund; 2022. Available: https://data.unicef.org/wp-content/uploads/2022/09/Kenya-Case-Study-9_22.pdf. Accessed: 21 June 2025.

[108] Njeru I Kareko D Kisangau N Langat D Liku N Owiso G Use of technology for public health surveillance reporting: Opportunities, challenges and lessons learnt from Kenya. BMC Public Health. 2020;20:1101. 10.1186/s12889-020-09222-2 32660509 PMC7359619

[109] Dehnavieh R Haghdoost A Khosravi A Hoseinabadi F Rahimi H Poursheikhali A The District Health Information System (DHIS2): A literature review and meta-synthesis of its strengths and operational challenges based on the experiences of 11 countries. Health Inf Manag. 2019;48:62–75. 10.1177/1833358318777713 29898604

[110] Numair T Harrell DT Huy NT Nishimoto F Muthiani Y Nzou SM Barriers to the digitization of health information: A qualitative and quantitative study in Kenya and lao PDR using a cloud-based maternal and child registration system. Int J Environ Res Public Health. 2021;18:6196. 10.3390/ijerph18126196 34201107 PMC8228682

[111] MEASURE Evaluation. How Kenya Monitors Health Information System Performance Findings from a Case Study. 2017. Available: https://www.measureevaluation.org/resources/publications/TR-17-208_en.html. Accessed: 21 June 2025.

[112] Moirongo RM Aglanu LM Lamshöft M Adero BO Yator S Anyona S Laboratory-based surveillance of antimicrobial resistance in regions of Kenya: An assessment of capacities, practices, and barriers by means of multi-facility survey. Front Public Health. 2022;10:1003178. 10.3389/fpubh.2022.1003178 36518572 PMC9742437

[113] Sheikh A Anderson M Albala S Casadei B Franklin BD Richards M Health information technology and digital innovation for national learning health and care systems. Lancet Digit Health. 2021;3:e383–96. 10.1016/S2589-7500(21)00005-4 33967002

[114] GOV.UK. The future of healthcare: our vision for digital, data and technology in health and care. 17 October 2018. Available: https://www.gov.uk/government/publications/the-future-of-healthcare-our-vision-for-digital-data-and-technology-in-health-and-care/the-future-of-healthcare-our-vision-for-digital-data-and-technology-in-health-and-care. Accessed: 19 May 2023.

[115] National Health Service. Principles for supporting high quality consultations by video in general practice during COVID-19. London, UK: National Health Service; 2020. Available: https://www.england.nhs.uk/coronavirus/wp-content/uploads/sites/52/2020/03/C0479-principles-of-safe-video-consulting-in-general-practice-updated-29-may.pdf%0Ahttps://ejournal.poltektegal.ac.id/index.php/siklus/article/view/298%0Ahttp://repositorio.unan.ed. Accessed: 21 June 2025.

[116] Central Office of Information. Report of the Second Phase of the Independent Review of NHS Pathology Services in England. London, UK: Central Office of Information; 2008. Available: https://webarchive.nationalarchives.gov.uk/ukgwa/20130124044941/http://www.dh.gov.uk/prod_consum_dh/groups/dh_digitalassets/%40dh/%40en/documents/digitalasset/dh_091984.pdf. Accessed: 19 June 2025.

[117] Caputo M [The modernisation of pathology and laboratory medicine in the UK: Networking into the future]. Riv Ital Med Lab. 2008;4:209–10. Italian. PMC2423316 18566667

[118] World Health Organization. Kenya national action plan on antimicrobial resistance: Review of progress in the human health sector – Antimicrobial resistance policy information and action brief series. Geneva, Switzerland: World Health Organization; 2022. Available: https://iris.who.int/bitstream/handle/10665/364530/9789240062689-eng.pdf?sequence=1. Accessed: 19 June 2025.

[119] Readkong. Uganda National Health Laboratory Services Policy II - THE REPUBLIC OF UGANDA MINISTRY OF HEALTH - September, 2016. 2016. Available: https://www.readkong.com/page/uganda-national-health-laboratory-services-policy-ii-the-7581886. Accessed 15 March 2023.

[120] SYNLAB. NHS Hub-and-Spoke Pathology Networks. 2020. Available: https://humanmedicine.synlab.co.uk/nhs-hub-and-spoke-pathology-networks/. Accessed: 19 May 2023.

[121] Oliwa JN Maina J Ayieko P Gathara D Kathure IA Masini E Variability in distribution and use of tuberculosis diagnostic tests in Kenya: A cross-sectional survey. BMC Infect Dis. 2018;18:328. 10.1186/s12879-018-3237-z 30012092 PMC6048895

[122] Oliwa JN Nzinga J Masini E van Hensbroek MB Jones C English M Improving case detection of tuberculosis in hospitalised Kenyan children—employing the behaviour change wheel to aid intervention design and implementation. Implement Sci. 2020;15:102. 10.1186/s13012-020-01061-4 33239055 PMC7687703

[123] Health Business. MoH to scale up numbers of GeneXpert® system from Cepheid – Health Business. 5 April 2022. Available: https://healthbusiness.co.ke/6274/moh-to-scale-up-numbers-of-genexpert-system-from-cepheid/. Accessed: 15 March 2023.

[124] Nalugwa T Shete PB Nantale M Farr K Ojok C Ochom E Challenges with scale-up of GeneXpert MTB/RIF® in Uganda: a health systems perspective. BMC Health Serv Res. 2020;20:162. 10.1186/s12913-020-4997-x 32131814 PMC7057496

[125] Ntinginya NE Kuchaka D Orina F Mwebaza I Liyoyo A Miheso B Unlocking the health system barriers to maximise the uptake and utilisation of molecular diagnostics in low-income and middle-income country setting. BMJ Glob Health. 2021;6:e005357. 10.1136/bmjgh-2021-005357 34429298 PMC8386239

[126] National Health Service. Neonatology GIRFT Programme National Specialty Report. England, UK: National Health Service; 2022. Available: https://gettingitrightfirsttime.co.uk/wp-content/uploads/2025/01/Neonatology-national-report-09-12i-FINAL.pdf. Accessed: 19 June 2025.

[127] National Health Service. Neonatology GIRFT Programme National Specialty Report. England, UK: National Health Service; 2022. Available: https://gettingitrightfirsttime.co.uk/wp-content/uploads/2025/01/Neonatology-national-report-09-12i-FINAL.pdf. Accessed: 19 June 2025.

[128] Ministry of Health of Uganda. Uganda National Supply Chain Assessment. Kampala, Uganda; Ministry of Health of Uganda; 2018. Available: https://health.go.ug/sites/default/files/Uganda_NSCA_Report_FINAL.pdf. Accessed: 19 June 2025.

[129] Galukande M Maling S Kabakyenga J Nshaho J Oboke H Oonge B Equitable access to health professional training in Uganda: A cross-sectional study. Ann Glob Health. 2018;84:91–9. 10.29024/aogh.7 30873807 PMC6748279

[130] Ssengooba F, Kiwanuka SN. Health Workforce Developments: Challenges and Opportunities to secure Universal Health Coverage in Uganda. In: Ssengooba F, Kiwanuka SN, Rutebemberwa E, Ekirapa-Kiracho E, editors. Universal Health Coverage in Uganda. Kampala, Uganda: Makerere University; 2018. p. 245–271.

[131] Ministry of Health of Kenya. Kenya Health Workforce Report: The Status of Healthcare Professionals in Kenya, 2015. Nairobi, Kenya: Ministry of Health of Kenya; 2019. Available: https://taskforce.org/wp-content/uploads/2019/09/KHWF_2017Report_Fullreport_042317-MR-comments.pdf. Accessed: 19 June 2025.

[132] Mumbo HM Kinaro JW Assessment of quality and relevance of curricula development in health training institutions: A case study of Kenya. Hum Resour Health. 2015;13:67. 10.1186/s12960-015-0048-9 26268602 PMC4535832

[133] Organisation for Economic Co-operation and DevelopmentMedical graduates. 2004. Available: https://www.oecd.org/en/data/indicators/medical-graduates.html?oecdcontrol-00b22b2429-var3=2004. Accessed: 10 September 2024.

[134] Ferreira T Collins AM Feng O Samworth RJ Horvath R Career intentions of medical students in the UK: A national, cross-sectional study (AIMS study). BMJ Open. 2023;13:e075598. 10.1136/bmjopen-2023-075598 37699638 PMC10496670

[135] The Health Foundation. The GP shortfall in numbers - The Health Foundation. 2022. Available: https://www.health.org.uk/features-and-opinion/features/the-gp-shortfall-in-numbers#:~:text=In%202021%2F2022%20there%20was,in%20post%20in%202021%2F22. Accessed: 18 May 2023.

[136] National Health Service. Diagnostics: Recovery and Renewal. England, UK: National Health Service; 2020. Available: https://www.england.nhs.uk/wp-content/uploads/2020/10/BM2025Pu-item-5-diagnostics-recovery-and-renewal.pdf. Accessed: 19 June 2025.

[137] Legislation.gov.uk. Coronavirus Legislation. 2020. Available: https://www.legislation.gov.uk/coronavirus. Accessed: 3 August 2023.

[138] China Global Television Network. Uganda approves private laboratories to carry out COVID-19 tests. 2023. Available: https://africa.cgtn.com/uganda-approves-private-laboratories-to-carry-out-covid-19-tests/. Accessed: 16 August 2024.

[139] Ministry of Health. Kenya Health Public Private Collaboration Strategy. 2020. Available: http://guidelines.health.go.ke:8000/media/The_Kenya_Health_Public_Private_Collaboration_Strategy_2020.pdf. Accessed: 3 August 2023.

[140] Ministry of Health of Kenya. Kenya Health Facility Census Report. Nairobi, Kenya: Ministry of Health of Kenya; 2023. Available: https://www.health.go.ke/sites/default/files/2024-01/Kenya%20Health%20Facility%20Census%20Report%20September%202023.pdf. Accessed: 19 June 2025.

[141] Bahati F English M Sayed S Horton A Odhiambo OA Samatar A Information asymmetry in the Kenyan medical laboratory sector. Glob Health Action. 2021;14:1964172. 10.1080/16549716.2021.1964172 34445946 PMC8405108

[142] Initiative for Social and Economic Rights. Profiteering Off a Pandemic Private Sector and Health Services. Kampala, Uganda: Initiative for Social and Economic Rights; 2021. Available: https://iser-uganda.org/wp-content/uploads/2022/03/Profiteering_off_a_pandemic.pdf. Accessed: 19 June 2025.

[143] Tashobya CK, Ogora VA, Kiwanuka SN, Mutebi A, Musila T, Byakika S, et al. Decentralisation and the Uganda Health System: What can we Learn from Past Experiences to Facilitate the Achievement of Universal Health Coverage? In: Ssengooba F, Kiwanuka SN, Rutebemberwa E, Ekirapa-Kiracho E, editors. Universal Health Coverage in Uganda. Kampala, Uganda: Makerere University; 2018. p. 117–150.

[144] The Guardian. Record rise in people using private healthcare amid NHS frustration. 2023. https://www.theguardian.com/society/2023/may/24/record-rise-in-people-using-private-healthcare-amid-nhs-frustration. Accessed: 3 August 2023.

[145] Doyle Y Bull A Role of private sector in United Kingdom healthcare system. BMJ. 2000;321:563–5. 10.1136/bmj.321.7260.563 10968825 PMC1118448

[146] Mugasha R Kwiringira A Ntono V Nakiire L Ayebazibwe I Kyozira C Scaling Up and Enhancing the Functionality of the Electronic Integrated Diseases Surveillance and Response System in Uganda, 2020-2022: Description of the Journey, Challenges, and Lessons Learned. JMIR Public Health Surveill. 2025;11:e59783. 10.2196/59783 40228314 PMC12011313

[147] Sapkota S Dhakal A Rushton S Van Teijlingen E Marahatta SB Balen J The impact of decentralisation on health systems: a systematic review of reviews. BMJ Glob Health. 2023;8:e013317. 10.1136/bmjgh-2023-013317 38135299 PMC10749071

[148] Sakala M Johnson C Chirombo J Sacks JA Baggaley R Divala T COVID-19 self-testing: Countries accelerating policies ahead of WHO guidelines during pandemics, a global consultation. PLOS Glob Public Health. 2024;4:e0002369. 10.1371/journal.pgph.0002369 38498477 PMC10947679

[149] Renzaho AMN Challenges Associated With the Response to the Coronavirus Disease (COVID-19) Pandemic in Africa—An African Diaspora Perspective. Risk Anal. 2021;41:831–6. 10.1111/risa.13596 32949030 PMC7537049

[150] Association for Diagnostics & Laboratory Medicine. How Specimen Referral Systems Can Improve. 2017. Available: https://www.aacc.org/cln/cln-stat/2017/april/20/how-specimen-referral-systems-can-improve-global-health. Accessed: 25 July 2023.

[151] Satta G Edmonstone J Consolidation of pathology services in England: Have savings been achieved? BMC Health Serv Res. 2018;18:862. 10.1186/s12913-018-3683-8 30442126 PMC6238267

[152] Joloba M Mwangi C Alexander H Nadunga D Bwanga F Modi N Strengthening the Tuberculosis Specimen Referral Network in Uganda: The Role of Public-Private Partnerships. J Infect Dis. 2016;213:S41–6. 10.1093/infdis/jiw035 27025697 PMC4914746

[153] Alamo T Reina DG Millán Gata P Preciado VM Giordano G Data-driven methods for present and future pandemics: Monitoring, modelling and managing. Annu Rev Control. 2021;52:448–64. 10.1016/j.arcontrol.2021.05.003 34220287 PMC8238691

[154] Olu O Muneene D Bataringaya JE Nahimana MR Ba H Turgeon Y How Can Digital Health Technologies Contribute to Sustainable Attainment of Universal Health Coverage in Africa ? A Perspective. Front Public Health. 2019;7:341. 10.3389/fpubh.2019.00341 31803706 PMC6873775

[155] Konnick EQ Laser J Weck KE The Role of Clinical Laboratories in Emerging Pathogens - Insights from the COVID-19 Pandemic. JAMA Health Forum. 2021;2:e213154. 10.1001/jamahealthforum.2021.3154 36218899

[156] National Health Laboratory and Diagnostic Services. Equipment and Supplies. Available: https://cphl.go.ug/nhlds-units. Accessed: 1 August 2023.

[157] Wadge H, Roy R, Sripathy A, Prime M, Carter A, Fontana G, Marti J, Chalkidou K. Evaluating the Impact of Private providers on health and health systems. London, UK: Imperial College London; 2017. Available: https://www.imperial.ac.uk/media/imperial-college/institute-of-global-health-innovation/Impact-of-private-providers-on-health-and-health-systems.pdf. Accessed: 21 June 2025.

[158] Westmore B Policy incentives for private innovation and maximising the returns. OECD J Econ Stud. 2014;2013:121–63.

[159] Mills A Brugha R Hanson K McPake B What can be done about the private health sector in low-income countries? Bull World Health Organ. 2002;80:325–30. 12075370 PMC2567770

